# The Effect of Applying Direct Observation of Procedural Skills (DOPS) on Nursing Students’ Clinical Skills: A Randomized Clinical Trial

**DOI:** 10.5539/gjhs.v7n7p17

**Published:** 2015-03-26

**Authors:** Habibi Hengameh, Raiesifar Afsaneh, Khaghanizade Morteza, Mahmudi Hosein, Seyed Mazhari Marjan, Abbas Ebadi

**Affiliations:** 1 Faculty of Nursing, Aja University of Medical Science, Tehran, Iran; 2 Faculty of Nursing and Midwifery, Tehran University of Medical Sciences, Tehran, Iran; 3 Behavioral Sciences Research Center (BSRC), Baqiyatallah University of Medical Sciences, Tehran, Iran; 4 Faculty of Nursing, Baqiyatallah University of Medical Sciences, Tehran, Iran

**Keywords:** DOPS, direct observation of procedural skills, clinical evaluation, nursing education

## Abstract

**Background:**

Remarkable advances in educational measurement have proved need to the implementation of modern and appropriate methods of clinical evaluation. This study was carried out to compare the effect of applying direct observation procedural skills and routine evaluation method on clinical skills of nursing students.

**Methods:**

This randomized clinical trial was conducted on students of Nursing Army College, Tehran, Iran. After obtaining approval from the Ethics Committee of the Baqiyatallah University of Medical Sciences Research Deputy, all nursing students and instructors who agreed to participate in this study sign the informed consent. The participants were randomly assigned into intervention and control groups. After the teachers were trained and an inter-raters reliability test was conducted, evaluation was performed through DOPS in the intervention group while the control groups were evaluated through the routine method. Assessment checklists for two procedures (Intra venous catheterization and change dressing) were valid and reliable. Finally data were analyzed through descriptive and analytical statistics (Chi-square, t-test, Repeated Measure ANOVA) using SPSS version 16.

**Results:**

No significant difference was observed between the two groups in terms of demographic variables (P>0.05), but a significant difference was observed between intervention and control scores (P=0.000). In other words, application of DOPS has improved clinical skills of the students significantly.

**Conclusion:**

Using this new method improved the students’ scores in clinical procedures implementation; therefore, we suggest that nursing colleges apply this evaluation method for clinical education.

## 1. Introduction

Clinical education is a set of facilitators of learning activities in a clinical environment. The purpose of such education is to create measurable changes in students clinical practice (Abedini, Aghamolaee, Jome Zadeh, & Kamjo, 2009). More than half of the educational goals of the nursing course are devoted to clinical education (Ebrahimi & Oudi, 2005). Nursing education managers recognize clinical education as the most important part of nursing education (Rahimi, 2005). Clinical education is one of the strategies for developing nurses’ clinical competency (Zareiyan Jahromi, 2005). In this regard, one of the important and challenging issues in clinical education is students’ evaluation in the field. Evaluation is the most important duty which the faculties are confronted with (Crossley Humphris, & Jolly, 2002). Through an appropriate evaluation method, possible strengths and weaknesses of education can be enumerated. The next step to the recognition of the pros and cons of the system positive aspects can be improved and shortcomings eradicated, this would be another step toward renovating of the entire educational system (Smith-Strøm & Nortvedt, 2008). Effective evaluation creates motivation in students, and also it help the educator with evaluating their activities (Franko et al., 2008) and if it is accompanied with feedback, it will be effective on promoting the learning of individual skill (Bari, 2010).

As a result of constant changes of approaches in clinical education, the necessity of implementing appropriate new methods of evaluation has become more succinct than before (Noohi & Haghdoost, 2008). Feedback oriented clinical evaluation methods will promote learning in addition to evaluating the difficult issues in traditional students’ evaluation (Kariman, 2010). To carry out evaluation in clinical environments, different methods can be used such as performance observation, 360 degree evaluation, objective structured clinical examination, Mini-clinical Evaluation Exercise (Mini-CEX) and Direct Observation of Procedural Skills (DOPS). This latter one, DOPS, is a method for evaluation and providing feedback pertaining to practical skills such as Intravenous catheterization, ECG taking, change dressing and etc. In this method, observation and clinical skill are performed through adopting the procedure by a student on a real patient (Crossley et al., 2002). DOPS is held at three levels with specified time intervals. At the end of each level, evaluator observes the student during performing the procedure then provides feedback to student and mentions their strengths and weaknesses. At the end of the third level, the evaluator rates the students’ performance by using a structured checklist and gives them the feedback (Malekanrad, 2006).

Although clinical skill have important role in medical education, success of educated medical groups follow their memories to some extent. According to the literature in the most of clinical courses, evaluation methods do not include efficiency in evaluation of clinical skills and students’ function as well as inappropriateness with educational objectives (Nouhi & Foroud, 2003; Rushforth, 2007; Asefzadeh, 2000; Schoonheim-Klein Walmsley, Habets, Van Der Velden, & Manogue, 2005). This is while practical skill have had the main role in medical education and mental knowledge is of secondary importance (Noohi & Haghdoost, 2008). Therefore the current study was conducted to assess the effect of DOPS method as a new evaluation method on promotion of nursing students’ clinical skills.

## 2. Methods

This is a randomized clinical study. An approval was obtained from the Ethics Committee of the Baqiyatallah University of Medical Sciences Research Deputy. The purpose and methods of the study were explained for the participants, both the educators and students, and then informed consent was obtained. All nursing students, who were passing medical-surgical and intensive courses in tow hospitals fields, were allocated into two intervention and control groups randomly. Ten clinical instructors who desired to participate and passing the new evaluation methods workshop were placed into two intervention and control groups at random. The students and the instructors were allowed to leave the study whenever they would not prefer to continue. Considered procedures in this study included change dressing and IV catheterization. The study consisted of two general phases of A) preparation and training the instructors and B) implementation of evaluation plan. In the first phase, the required training protocol was prepared for DOPS method then the intervention instructors group was trained within a four-hour workshop.

In the second phase, control group was evaluated based on the routine evaluation method of Army Nursing College and intervention group was evaluated based on the DOPS. The routine method of Nursing College includes a subjective judgment of an instructor about general skills of the student during their clinical course hence the scoring. In intervention group, clinical activities of students (performance of the aforementioned procedures) were evaluated through the DOPS based on the checklists. The evaluation steps in intervention group took place as follow:

A) First stage test: (observation of the skills for 15 minutes and giving feedback for 5 minutes).
B) Second stage test: repeating the first test after two weeks (emphasis on providing feedback on the students’ strength and weakness).
C) Third stage test: repeating the first stage test after four weeks and giving the final scores to the student.

In the control group, two procedures were evaluated only in one stage as suggested by the routine. Final evaluation of students in each group was performed based on the checklists prepared by the researchers. A zero score represents observing no appropriate behavior by a student, one is less than expectation, 2 bordering, 3 expected, and 4 is excellent. For assessment of psychometric properties of the checklists, after an in-depth library and digital search to find out how the procedures is to be implemented, an initial draft for each procedures was prepared. Next, the draft was submitted to 10 experienced faculty members in the relevant clinical field for content validity assessment. According to the Low-shed table, content validity rate (CVR) was 0.62, based on which, the questions that obtained good points were selected. In order to assess the content validity index (CVI) with relevant formulation, the questions scored more than 0.79 considered as appropriate and those with scores between 0.70 and 0.79 were later revised by experts. The questions scored less than 0.70 were rejected. To determine the inter rater reliability, two methods of equivalence and inter rater reliability were used. Three teachers observed and evaluated five students for each procedure simultaneously; then, inter rater reliability between assessors was measured by ICC (Intraclass correlation coefficient) and each item was evaluated by Kappa coefficient test; Kappa coefficient was 0.6 and ICC equaled 0.5. Finally, the data was analyzed by the SPSS software version 16 within descriptive statistic tests (Frequency, mean and standard deviation) and analytical statistic tests such as Kolmogorov Smirnov, Chi-square, independent t-test, Repeated Measure ANOVA, Pearson correlation coefficient based on the type of variable.

## 3. Results

Homogeneity of the two groups of age and the total last terms means tested by independent t-test, gender, marital status and distribution of students in hospital units through Chi-square test and there was no meaningful difference in two groups (Table 1).

**Table 1:** Distribution of Demographic Variables in Intervention and Control Groups

Demographic variables		Intervention group	Control group	Test

		Mean± SD	Mean± SD	
Age(year)		23±0.84	22.8±0.58	T=-1.15 P=0.25 Df=68

Total average		15.9±1.59	16.19±1.42	T=-0.8 P=0.42 Df=68

		Frequency (%)	Frequency (%)	

Gender	Female	54.3	60	X^2^= 0.232 P= 0.62

	male	45.7	40	

Marital status	Married	31.4	45.7	X^2^= 1.5 P= 0.22

	Single	68.6	54.3	

The independent t-test indicates a significant difference in resulted scores from the two different evaluation methods (Table 2). Comparison of DOPS scores in three steps shows there is an increasing trend from step one to step three in both procedures [IV catheterization(p=0.000), change dressing(p<0.014)]. A significant correlation was observed between total students means in last semester and score of IV catheterization procedure (r=0.23, p<0.016) by using Pearson correlation coefficient.

**Table 2:** Comparison of Tow Procedures Final Scores in Intervention and Control Groups

Procedure	Intervention group (Mean± SD)	Control group (Mean± SD)	Independent sample t-test
IV catheterization	112.34±10.24	86.08±10.92	T=-10.373 Df=68 P=0.000
Change dressing	92.22±8.15	66.62±9.82	T=-11.858 Df=68 P=0.000

## 4. Discussion

This study was carried out in order to compare the effect of DOPS against the routine method on clinical skills promotion of nursing students. The results indicate that the DOPS test is more effective on skills level promotion of the nursing students in comparison with the traditional evaluation method. These findings were confirmed by the Shahgheibi et al. (2009) study conducted to assess the DOPS effect on the evaluation of clinical skills of internship course students in maternity units. In their study students’ mean scores which were evaluated through DOPS had significant difference in comparison with the control group (Shahgheibi Sh, 2009). In the present study, by performing DOPS in three stages, improvement of the students’ scores and the development of their skills level were seen. The reason for this development can be attached to more practice in implementation of procedure and taking tests in several stages and giving feedback in each stage. Bazrafkan (2009) study indicated that the students’ scores in DOPS test had normal distribution and 87.6% of students earned acceptable scores (Bazrafkan, 2009). Also in this study there was an ascending trend in two procedures’ scores in intervention group. Wang et al. (2007) cohort study with the purpose of evaluating the clinical skills development of nurses through DOPS and mini-CEX, indicated that these evaluation methods cause development in skills, learning strategies, and attitude toward hospital, assessment the ability and performance and improving the quality of interventions in nursing practices (Wang and Lin, 2007). In this study, no significant correlation was observed between students’ demographic variables and clinical skills scores but total average of passed last courses had significant correlation with IV catheterization procedure score also. In Salimi et al. (2005) study no meaningful difference was seen between male and female students’ clinical skill but there was a significant difference between average of last semester and clinical skills mean scores in intensive units (Salimi, 2005). Therefore, it seems that better theoretical background and higher average have direct effect on skill learning and increasing the level of the skill. Sinclair and Cleland (2007) in their study concluded that male medical students, who are weak in theory, may not use feedback for assessing their learning experiences. Female and cleverer students follow feedbacks to improve their status (Sinclair & Cleland, 2007). As it can be noticed, in different studies, there are similar results about significant difference between final score of DOPS and the traditional evaluation method. Compared with new methods, such as the OSCE which evaluation done in artificial conditions or log-books which measures the quantity of procudures or acts, DOPS have more validity. Sahebalzamani et al. (2012) study showed that a high percentage of studied teachers and students are satisfied or very satisfied with evaluation with DOPS. Thay argued that the students are satisfied when assessed by those with more knowledge level (Sahebalzamani & Jahantigh, 2012).

## 5. Conclusion

This study showed that using DOPS improved the students’ scores in clinical procedures implementation. Thus, it is recommended that in various courses or procedure evaluation, the traditional methods be replaced with the DOPS. These new evaluation techniques could reduce the most important educational problem that is theory-practice gap especially in medical sciences fields, which are more sensitive fields. The limitations of this study were instructors’ resistance and lack of inclination for the new evaluation methods. Also, unfortunately in this study, the possibility of hawthorn effect from some teachers was not probed. Besides, due to the nature of the study no pretest could be taken by the participants.
